# Genomic taxonomy of vibrios

**DOI:** 10.1186/1471-2148-9-258

**Published:** 2009-10-27

**Authors:** Cristiane C Thompson, Ana Carolina P Vicente, Rangel C Souza, Ana Tereza R Vasconcelos, Tammi Vesth, Nelson Alves, David W Ussery, Tetsuya Iida, Fabiano L Thompson

**Affiliations:** 1 Laboratory of Molecular Genetics of Microrganims, Oswaldo Cruz Institute, FIOCRUZ, Rio de Janeiro, Brazil; 2 National Laboratory for Scientific Computing, Department of Applied and Computational Mathematics, Laboratory of Bioinformatics, Av. Getúlio Vargas 333, Quitandinha, 25651-070, Petropolis, RJ, Brazil; 3 Center for Biological Sequence Analysis, Department of Biotechnology, Building 208, The Technical University of Denmark, DK-2800 Kgs. Lyngby, Denmark; 4 Department of Genetics, Institute of Biology, Federal University of Rio de Janeiro, UFRJ, Brazil; 5 Laboratory of Genomic Research on Pathogenic Bacteria, International Research Center for Infectious Diseases, Research Institute for Microbial Diseases, Osaka University, Suita, Osaka 565-0871, Japan

## Abstract

**Background:**

Vibrio taxonomy has been based on a polyphasic approach. In this study, we retrieve useful taxonomic information (*i*.*e*. data that can be used to distinguish different taxonomic levels, such as species and genera) from 32 genome sequences of different vibrio species. We use a variety of tools to explore the taxonomic relationship between the sequenced genomes, including Multilocus Sequence Analysis (MLSA), supertrees, Average Amino Acid Identity (AAI), genomic signatures, and Genome BLAST atlases. Our aim is to analyse the usefulness of these tools for species identification in vibrios.

**Results:**

We have generated four new genome sequences of three *Vibrio *species, *i*.*e*., *V. alginolyticus *40B, *V*. *harveyi*-like 1DA3, and *V*. *mimicus *strains VM573 and VM603, and present a broad analyses of these genomes along with other sequenced *Vibrio *species. The genome atlas and pangenome plots provide a tantalizing image of the genomic differences that occur between closely related sister species, *e*.*g*. *V. cholerae *and *V. mimicus*. The vibrio pangenome contains around 26504 genes. The *V. cholerae *core genome and pangenome consist of 1520 and 6923 genes, respectively. Pangenomes might allow different strains of *V. cholerae *to occupy different niches. MLSA and supertree analyses resulted in a similar phylogenetic picture, with a clear distinction of four groups (*Vibrio *core group, *V. cholerae-V. mimicus*, *Aliivibrio *spp., and *Photobacterium *spp.). A *Vibrio *species is defined as a group of strains that share > 95% DNA identity in MLSA and supertree analysis, > 96% AAI, ≤ 10 genome signature dissimilarity, and > 61% proteome identity. Strains of the same species and species of the same genus will form monophyletic groups on the basis of MLSA and supertree.

**Conclusion:**

The combination of different analytical and bioinformatics tools will enable the most accurate species identification through genomic computational analysis. This endeavour will culminate in the birth of the online genomic taxonomy whereby researchers and end-users of taxonomy will be able to identify their isolates through a web-based server. This novel approach to microbial systematics will result in a tremendous advance concerning biodiversity discovery, description, and understanding.

## Background

Taxonomy is at the basis of the biological sciences, being one of its oldest branches. It deals with identification, classification (*i*.*e*. creation of new taxa) and nomenclature. In the early 1970s, a seminal work done by Colwell put forward the concept of polyphasic taxonomy that is currently still in use [1]. According to this concept, in order to achieve the most natural classification system, there should be an integration of information from the molecular to the ecological levels. DNA-DNA hybridization (DDH) data had a pivotal role to infer the species boundaries in the polyphasic taxonomy. Indeed DDH proved to be the most powerful tool to unambiguously identify prokaryotes, providing for the first time, a reliable means of categorizing microbes. However, this technique suffers from various limitations, including the need to include reference strains in each new experiment. In addition, it is not portable and requires special facilities available in a limited number of international laboratories. The introduction of 16S rRNA analysis as an alternative method for use in taxonomy allowed the development of an evolutionary framework [2,3]. Boundaries for species identification and a species definition were set on the basis of extensive empirical data [4]. A bacterial species is defined as a group of strains (including the type strain), having > 70% DDH similarity, < 5°C ΔT_m_, < 5% mol G+C difference of total genomic DNA, > 97% 16S rRNA identity [4,5].

The bacterial species definition is pragmatic and operational, aiming at the establishment of a rapid, reliable, reproducible, and useful taxonomic framework, based on microbial evolution, for a variety of applications (*e*.*g*. medicine and agriculture) [6]. This polyphasic definition is a consensus in microbiology, although it is not based on a concept (i.e. the biological processes behind speciation and species). It is crucial to highlight that the current polyphasic framework does not question if this definition corresponds to a biological reality [7]. Within the framework of polyphasic taxonomy, strains of the same species have similar phenotypes (*e*.*g*. expression of different types of enzymes, ability to using different types of compounds as energy source, and growth in different temperatures and concentrations of acid and salt), genotypes (*e*.*g*. rep-PCR and AFLP), and chemotaxonomic features (*e*.*g*. FAME and polyamines), forming distinguishable tight groups [7]. Ideally, these groups should be readily identifiable and differentiated from closely related species. However, currently there is not a consensus on the definition of a bacterial genus [7].

The most recent ideas on the species concepts corroborate polyphasic taxonomy [8]. According to these authors, a bacterial species concept is a framework that explains how bacterial strains share common features and how they maintain genomic cohesion. The cohesion is observed as recurrent patterns or groups of strains that are recognized in nature and are termed species for practical reasons. However, the biological process giving rise and maintaining cohesion of these groups is what matters in a species concept. Homologous recombination appears to be a major force leading to genomic cohesion of strains of the same species. It is more frequent between strains of the same species than between strains of different species simply because this genetic process depends on sequence similarity. Horizontal gene transfer (HGT) between unrelated strains would cause an increase in phenotypic variation, but would not be frequent enough to hamper the formation and recognition of species. An alternative species concept is the stable ecotype in which cohesion of bacterial strains of a given species is maintained by accumulation of advantageous mutations and periodic purging of allelic variability [9]. In contrast with the polyphasic species definition which is widely accepted, there is not a consensus on a bacterial species concept. It is of course possible that one single concept is not sufficient to explain the complexity of bacterial diversity. In addition, mutation, homologous recombination and HGT may be detected at varying levels in a single strain, making the scenario even more complex.

Whole microbial genome sequencing studies launched microbial taxonomy into a new era, with the possibility of establishing sistematics on the basis of complete genomes [10]. How does one go about using whole genome sequences (WGS) for establishing a genomic taxonomy? And more specifically, how can one taxonomically define and identify species by means of WGS? WGS may contain taxonomic information in the form of gene content, genome wide signatures, phylogenetic markers, amino acid identity and overall genetic composition that might be useful for building novel taxonomic schemes [11]. Pioneer computational and mathematical studies performed in the 1990s suggested that genomes contain species-specific signatures [12]. Genome signature is a compositional parameter reflecting the dinucleotide relative abundance, which is similar between closely related species, and dissimilar between non-related species. Genome signatures appear to allow the identification of isolates and metagenomes into known species [13,14]. Whole genome sequences also permit the reconstruction of more robust taxonomic trees (*i*.*e*. supertrees) based on all genes of the core genome [15-17]. A good congruence was obtained by the traditional 16S rRNA based trees and the novel supertree methods [18] proposed that the average amino acid identity (AAI) could be used to distinguish closely related sister species. Subsequently, a close relationship between DDH and AAI was shown [19]. Some studies have suggested that the effective number of codons (Nc) could also be a species-specific marker [20].

In *Vibrios*, the birth of the genomic taxonomy occurred with a series of papers that attempted to use multilocus sequence analysis (MLSA) [21-26]. These studies allowed the establishment of rapid and powerful identification systems through the internet. Currently there are MLSA schemes for most of the human pathogens available for free access in the internet. Establishing a universal MLSA will not be possible though. Studies accomplished so far have shown that the resolution of different markers varies according to the taxonomic groups. For instance, the *recA *gene is very useful to differentiate closely related species of *Burkholderia *[27], but it is not appropriate for vibrios [24]. Clearly, genes have different molecular clocks in different microbes, indicating the need of a multigene approach. With the advent of ultra-rapid genome sequencing, it is now possible to sequence one almost complete microbial genome in less than a day [28,29]. The new generation of DNA sequencers will enable sequencing of more than a dozen prokaryotic genomes in less than an hour, possibly making it cheaper and faster to sequence a whole genome than several genes for MLSA. In future, MLSA might be used simply as a rapid screen methodology [30].

Vibrios are an excellent test model for genomic taxonomy because they are ubiquitous in the marine environment, associated with a wide range of marine life (some species such as *V. cholerae*, *V. parahaemolyticus *and *V. vulnificus *cause serious disease in man) and experiencing a variety of environmental conditions and selection forces, leading to high genomic plasticity [31,32]. Consequently, differentiation of sister species becomes very difficult. For instance, *V. cholerae *and *V. mimicus *have nearly indistinguishable phenotypes. Among the phenotypic tests used in the Bergey's manual, only sucrose fermentation and lipase activity may discriminate the two species. According to the most recent version of the Bergey's manual, a Vibrio species is defined as a group of strains forming small (0.5-0.8 × 1.4-2.6 μm) comma-shapped rods with polar flagella enclosed in a sheath, facultative anaerobic metabolism, capable of fermenting D-glucose and growth at 20°C [33]. Primarily aquatic, most species are oxidase positive, reduce nitrate to nitrite, require Na+ for growth, and ferment D-frutose, maltose, and glycerol. Each vibrio species is further identified by an array of over 100 phenotypic tests. There is not an operational definition for genera within the vibrios [33]. In our hands, vibrio species may be better defined on the basis of amplified fragment length polymorphism (AFLP) and MLSA [21,22,25,34]. Strains of the same species (including the type strain) share more than 60% mutual AFLP band pattern similarity and more than 95% similarity in MLSA (using the loci *rpoA, recA, pyrH, ftsZ, topA, mreB gyrB *and *gapA*). More importantly, strains of the same species and species of the same genus will form monophyletic groups on the basis of MLSA. This was the main argument used to propose the newly described genus *Aliivibrio *[35].

In order to test the feasibility of the genomic taxonomy in vibrios, several markers were analysed in a collection of 32 genomes, including four newly pyrosequenced genomes. Several *Vibrio *strains had the genome completely sequenced and are available on the web. Eleven *V. cholerae *and two *V. mimicus *genomes formed an ideal test case for taxonomy because of their close relatedness as sister species. These sister species have nearly identical 16S rRNA sequences and around 70% DDH. Disclosing species-specific patterns for the different genome-wide markers would reinforce their usefulness in prokaryotic taxonomy. The aim of this study was to extract taxonomic information from vibrio genome sequences by means of a detailed analysis of MLSA, supertree, Nc, AAI, genomic signatures, Genome BLAST atlas and pangenome plot that would allow species identification.

## Methods

### Genome sequence data

We used 32 genomes of vibrios in this study unless otherwise stated. The genomic sequences of 28 vibrios were obtained from the National Center for Biotechnology Information (NCBI) (Table 1). We have sequenced the genome of *V. alginolyticus *40B, *V*. *harveyi*-like 1DA3, and *V*. *mimicus *strains VM573 and VM603. *V. alginolyticus *40B and *V*. *harveyi*-like 1DA3 were isolated from Brazilian corals (*Mussismilia hispida *and *Phyllogorgia dilatata *in 2007 at the Abrolhos reef bank, respectively). *V. mimicus *VM573 (CT and TCP positive) was isolated from a patient with diarrhea in 1990s in the US, whereas *V. mimicus *VM603 was isolated from riverine water in the Brazilian Amazonia region in 1990s. These genomes were sequenced by the Roche-454 pyrosequencing method. Genomic DNA was extracted using the method of Pitcher [36]. The pyrosequencing technique was performed according to [37]. Briefly, genomic DNA was randomly sheared to small fragments and ligated to common adaptors. Single fragments were attached to beads in an emulsion. Amplification by PCR was done in the emulsion and produced ~10^7 ^copies of the fragments per bead. After removal of the emulsion, the beads were deposited on a fiber optic slide. The DNAs were sequenced using a pyrosequencing protocol. Sequencing of *V. mimicus *VM603 genome was performed on the prototype Roche 454 Genome Sequencer 20™ system, whereas sequencing of *V. alginolyticus *40B, *V*. *mimicus *VM573 and *V*. *harveyi*-like 1DA3 genomes was performed on a Roche 454 Genome Sequencer FLX™ system. The reads were assembled using the Newbler software of the 454/Pyrosequencing. These genomes were annotated automatically using the software SABIÁ [38] and have been deposited at DDBJ/EMBL/GenBank under the project accession number [GenBank:ACZB00000000] (*V. alginolyticus *40B), [GenBank:ACZC00000000] (*V*. *harveyi*-like 1DA3), [GenBank:ACYV00000000] (*V*. *mimicus *VM573) and [GenBank:ACYU00000000] (*V. mimicus *VM603). The version described in this paper is the first version. The genomes are also available online . The DNA G+C content of *V. alginolyticus *40B, *V*. *mimicus *VM573 and *V*. *harveyi*-like 1DA3 genomes was calculated using MEGA version 4.0 [39]. We used concatenated genomic sequences of the two chromosomes of vibrios for our analyses.

**Table 1:** Genomic features of the vibrios genomes.

**Organism**	**Accession no**.	**Genome size (nt)**	**G+C (mol%)**	**No. of CDS**	**%** **coding region**	Nc*
Aliivibrio salmonicida FLI1238	FM178379	3325164	39		77	48
Chromosome I	FM178380	1206461	38	2820	77	
Chromosome II				984		
Photobacterium profundum SS9						
Chromosome I	CR354531	4085304	41	3416	82	51
Chromosome II	CR354532	2237943	41	2006	80	
						
**Vibrio alginolyticus 40B**	ACZB00000000	5234286	45	4341	81	53
Vibrio alginolyticus 12G01	AAPS00000000	5160431	44	4732	86	53
Vibrio angustum S14^+^	AAOJ00000000	5101447	39	4558	84	48
Vibrio campbellii AND4	ABGR00000000	4255798	44	3935	85	53
Vibrio cholerae N16961	AE003852	2961149	47		87	52
Chromosome I	AE003853	1072315	46	2742	84	
Chromosome II				1093		
Vibrio cholerae 0395	CP000627	3024069	47		88	52
Chromosome I	CP000626	1108250	46	2742	86	
Chromosome II				1133		
Vibrio cholerae 1587	AAUR00000000	4137501	47	3758	82	52
Vibrio cholerae 2740-80	AAUT00000000	3945478	47	3771	87	52
Vibrio cholerae 623-39	AAWG00000000	3975259	47	3777	86	52
Vibrio cholerae B33	AAWE00000000	4026835	47	3677	83	53
Vibrio cholerae MAK757	AAUS00000000	3917446	47	3501	82	52
Vibrio cholerae MZO-2	AAWF00000000	3862985	47	3425	83	52
Vibrio cholerae MZO-3	AAUU00000000	4146039	47	3897	86	52
Vibrio cholerae NCTC8457	AAWD00000000	4063388	47	3975	86	53
Vibrio cholerae V52	AAKJ00000000	3974495	47	3815	86	52
Vibrio fischeri ES114^+^	CP000020	2897536	38			45
Chromosome I	CP000021	1330333	37	2586	86	
Chromosome II				1175	87	
Vibrio fischeri MJ11^+^						45
Chromosome I	CP001139	2905029	38	2590	86	
Chromosome II	CP001133	1418848	37	1254	87	
						
**Vibrio harveyi-like 1AD3**	ACZC00000000	5989646	46	4954	66	51
Vibrio harveyi ATCC BAA-1116	CP000789	3765351	45		85	53
Chromosome I	CP000790	2204018	45	3546	86	
Chromosome II				2374		
Vibrio harveyi HY01	AAWP00000000	5400985	45	4327	75	51
						
						
**Vibrio mimicus VM573**	ACYV00000000	4373300	46	3744	86	53
						
**Vibrio mimicus VM603**	ACYU00000000	4421792	46	3790	86	53
Vibrio parahaemolyticus RIMD2210633						
Chromosome I	BA000031	3288558	45	3080	86	52
Chromosome II	BA000032	1877212	45	1752	86	
Vibrio parahaemolyticus AQ3810	AAWQ00000000	5771228	45	5509	80	53
Vibrio shilonii AK1	ABCH00000000	5701826	43	5360	88	54
Vibrio sp Ex25	AAKK00000000	4844262	44	4240	84	53
Vibrio sp MED222	AAND00000000	4891901	43	4590	85	52
Vibrio splendidus 12B01	AAMR00000000	5596386	44	5231	85	53
Vibrio vulnificus CMCP6						
Chromosome I	AE016795	3281944	46	2915	83	53
Chromosome II	AE016796	1844853	47	1557	86	
Vibrio vulnificus YJ016	BA000037	3354505	46		87	53
Chromosome I	BA000038	1857073	47	3259	89	
Chromosome II				1696		

The genomes sequenced by this study are in bold. ^+^*Vibrio angustum *and *Vibrio fischeri *were reclassified as *Photobacterium angustum *[67] and *Aliivibrio fischeri *[35], respectively. *calculated using concatenated chromosome sequences.

### Genome BLAST Atlas, proteome matrix, and pangenome plot

The BlastAtlas plots were constructed as described previously [40,41]. The pangenome plot, and proteome matrix were constructed as described [42]. For building the atlas, the genomes were automatically annotated and were compared to the reference chromosome (*V. cholera *strain N16961 in this case). The BLAST matrix perl script performs an all-against-all BLAST comparison of genomes from multiple organisms. For every combination, a protein blast is carried out, finding all homologous proteins. For our purposes, we use the "50-50 rule", which requires both of the following characteristics: 1.) at least 50% of the query protein must overlap in the alignment, and 2.) at least 50% of the residues within the alignment must be identical. After the homologous proteins are identified, the proteins are clustered into protein families and the number of families containing proteins from both strains are counted. The fraction of these shared families out of the total number of families is the number reported in the BLAST matrix. Since the direction of comparison of the two organisms will give identical results under these conditions, one redundant half of the square matrix plot is left out. Thus, we use a triangular shaped diagram where the hypotenuse corresponds to the paralogs (red), which are the internal homologous proteins (*e.g*., repeated genes). Since this is a comparison of all the proteins in a genome, compared to the pan-genome, it is possible to see related organisms, in terms of their similar composition of gene families.

### 16S rRNA tree, Multilocus Sequence Analysis (MLSA) and Supertree approach

MLSA and supertree approach were based on the concatenated sequences of house-keeping genes [15,21]. The 16S rRNA gene sequences, the gene sequences used for MLSA (*i.e*. *ftsZ, gyrB, mreB, pyrH, recA, rpoA *and *topA*) and the gene sequences used for supertree (*i.e*. aminopeptidase P, *alaS, aspS, ftsZ, gltX, gyrB, hisS, ileS, infB, metG, mreB, pntA, pheT, pyrH, recA, rpoA, rpoB, rpsH*, signal recognition particle protein, threonyl-tRNA synthetase, *topA, valS *and 30S ribosomal protein S11) were obtained from the NCBI. The concatenated sequences were aligned by CLUSTALX. Phylogenetic analyses were conducted using MEGA version 4.0 [39] and PAUP version 4.0b10 [43]. The phylogenetic inference was based on the maximum-parsimony character method (MP), the neighbour-joining genetic distance method (NJ) [44], and the maximum likelihood method (ML). Distance estimations were obtained according to the Kimura-2-parameter for 16S rRNA gene and Jukes-Cantor [45] for MLSA and supertree for NJ. The program Modeltest was used to select the GTR+I+G as the model for MLSA and supertree and Tamura-Nei+I+G as the model for 16S rRNA in the ML analysis. The reliability of each tree topology was checked by 2000 bootstrap replications [46].

### Average amino acid identity (AAI)

The AAI was calculated according to [18]. Genes conserved between a pair of genomes were determined by whole-genome pairwise sequence comparisons using the BLAST algorithm release 2.2.5 [47]. For these comparisons, all protein-coding sequences (CDSs) from one genome were searched against the genomic sequence of the other genome. CDSs that had a BLAST match of at least 40% identity at the amino acid level and an alignable region with more than 70% of the length of the query CDS were considered as conserved genes [48]. This cutoff is above the twilight zone of similarity searches, where inference of homology is error prone due to low similarity between aligned sequences. Thus, query CDSs were presumably homologous to their matches. The genetic relatedness between a pair of genomes was measured by the average amino acid identity of all conserved genes between the two genomes as computed by the BLAST algorithm.

### Codon usage

Codon usage bias was calculated for each genome. The effective number of codons used in a sequence (Nc) [20], was calculated using CHIPS (EMBOSS). Nc values range from 20 (in an extremely biased genome where only one codon is used per amino acid) to 61 (all synonymous codons are used with equal probability) [20]. The rose plot of codon usage was constructed as described previously [42].

### Determination of dinucleotide relative abundance values

We determined the dinucleotide relative abundance value for each genome. Sequences were concatenated with their inverted complementary sequence using REVSEQ, YANK and UNION (EMBOSS). Mononucleotide and dinucleotide frequencies were calculated using COMPSEQ (EMBOSS). Dinucleotide relative abundances (ρ*_XY_) were calculated using the equation ρ*_XY _= f_XY_/f_X_f_Y _where f_XY _denotes the frequency of dinucleotide XY, and f_X _and f_Y _denote the frequencies of X and Y, respectively [12]. Statistical theory and data from previous studies [12,49] indicate that the normal range of ρ*_XY _is between 0.78 and 1.23. The difference in genome signature between two sequences is expressed by the genomic dissimilarity (δ*), which is the average absolute dinucleotide of relative abundance difference between two sequences. The dissimilarities in relative abundance of dinucleotides between both sequences were calculated using the equation described by [12]: δ*(f,g) = 1/16Σ|ρ*_XY _(f) - ρ*_XY _(g)| (multiplied by 1000 for convenience), where the sum extends over all dinucleotides.

## Results

### General features of the sequenced genomes

**Figure 1:**
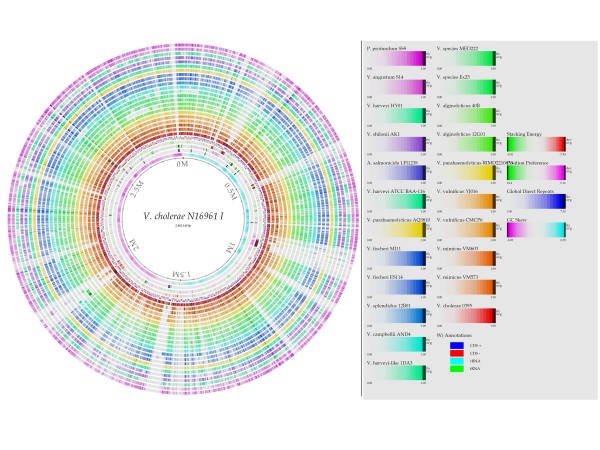
**Genome BLAST atlas. The chromosome I of vibrios**. The Atlas was constructed using the genome of *V. cholerae *N16961 as the reference strain on which the genes of the other strains are mapped. Genomic regions unique to this strain and not appearing in other vibrio strains are lightly colored. The position of the genes in the different replicons may not be the same.

**Figure 2:**
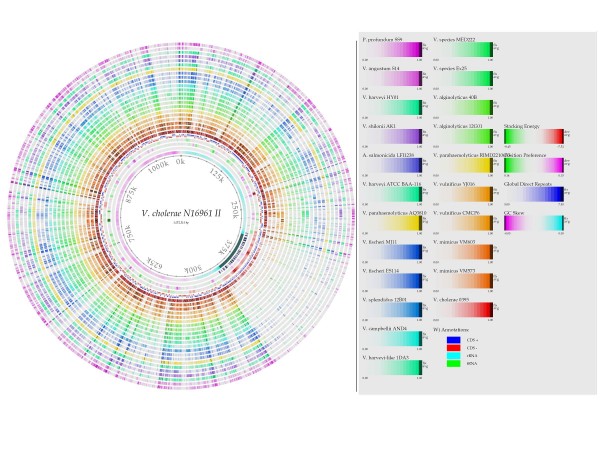
**Genome BLAST atlas. The chromosome II of vibrios**. The Atlas was constructed using the genome of *V. cholerae *N16961 as the reference strain on which the genes of the other strains are mapped. Genomic regions unique to this strain and not appearing in other vibrio strains are lightly colored. Notice the hyper-variable region (midpoint at 375 K) in the chromosome II of vibrios (the super-integron), corresponding to 1/6 of this replicon. The position of the genes in the different replicons may not be the same.

The new genomic sequences generated in this study for *V. alginolyticus *40B (ACZB00000000), *V. harveyi-like *1DA3 (ACZC00000000), *V. mimicus *strains VM573 (ACYV00000000), and VM603 (ACYU00000000), had 290, 229, 82 and 488 contigs with a total length of approximately 5,234,286, 5,989,646, 4,373,300 and 4,321,792 bp, respectively. The estimated coverage depth was 18, 22, 24 and 20×, respectively. The average GC content for the draft genomes were 45%, 46%, 46%, and 46%, respectively (Table 1). A first attempt to have a global visualization of the differences in gene content between the reference genome *V. cholerae *N16961 and the genomes of the other vibrios was obtained by the genome BLAST atlas which *per se *is not meant to be a taxonomic tool (Figure 1 and Figure 2). There are several regions (lightly colored) of low conservation throughout the chromosomes 1 and 2. In chromosome 2 there is a large region in the low right area that is poorly conserved within the other vibrios. This region corresponds to the superintegron [50]. We can observe in chromosome 1 and 2 that there are regions which contain genes that are conserved only in *V. cholerae*, missing in the other vibrio genomes. These regions might encode for some sort of environmental niche-specific genes. *V. cholerae *strains have little mutual gene content variation even in the hypervariable superintegron region (see chromosome II midpoint 375 Kb). The two *V. mimicus *genomes were the closest to the N16961 according to the atlas.

### Proteome BLAST

**Figure 3:**
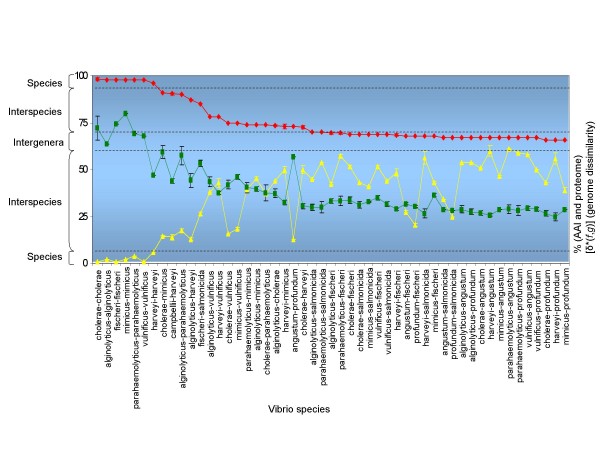
Taxonomic resolution of AAI, BLAST proteome and genome dissimilarity [δ*(*f*,*g*)] of vibrios. Mean and standard deviations. Red = AAI; Green = proteome; yellow = [δ*(*f*,*g*)]. The taxonomic resolution of AAI is down to the intergenera level, whereas [δ*(*f*,*g*)] has a resolution at interspecies level. The dashed lines delimit (p < 0.001) the different taxonomic levels for AAI and [δ*(*f*,*g*)] but not for the proteome. The proteome did not completely fit this figure (and dashed lines limits), showing some noise signal for *V. harveyi-V.harveyi*.

The BLAST proteome for all vibrio strains varied between 23.1% (*V. harveyi-P. profundum*) and 79.8% (*V. mimicus-V. mimicus*) similarity, whereas the percentage of paralogs varied between 1.8% (*V. mimicus-V. mimicus*) to 9.3% (*V. parahaemolyticus-V. parahaemolyticus*). The BLAST comparison indicated that *V. cholerae *genomes had mutual proteome identity at minimum 61.8% and at maximum 78.4% (Figure 3 and see Additional file 1; Table S1). The intraspecific proteome identity in *V. cholerae *varied between 61.8% and 78.3%, whereas the paralogs in *V. cholerae *genomes varied from 2.8% (99 proteins) to 3.8% (130 proteins). The sister species *V. cholerae*-*V. mimicus, V. parahaemolyticus-V. alginolyticus *and *V. harveyi-V. campbellii *had proteome identity at maximum 65.7%, 64.4% and 45%, respectively. The maximum proteome identity between the genera *Vibrio *and *Aliivibrio *was 38.6% (*i.e*. *V. splendidus *and *A. fischeri*), whereas the identity between *Vibrio *and *Photobacterium *was 31.8% (i.e. *V. splendidus *and *P. angustum*). *Aliivibrio *and *Photobacterium *had at maximum 32.3% identity.

### Phylogenetic reconstructions by 16S rRNA, MLSA and supertree

**Figure 4:**
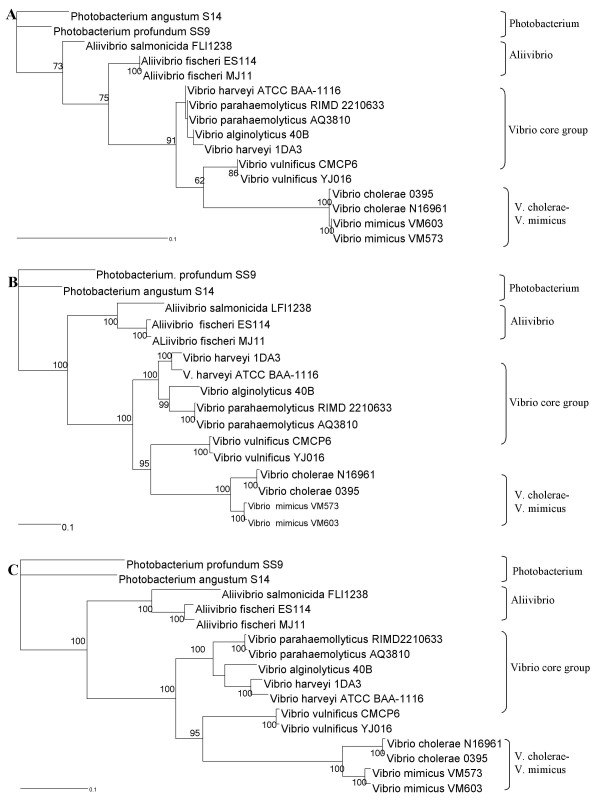
**A-C. Phylogenetic trees based on the maximum likelihood method using 16S rRNA gene**, MLSA (*i.e*. *ftsZ, gyrB, mreB, pyrH, recA, rpoA and topA*; 10,141 bp), and supertree (*i.e*. aminopeptidase P, *alaS, aspS, ftsZ, gltX, gyrB, hisS, ileS, infB, metG, mreB, pntA, pheT, pyrH, recA, rpoA, rpoB, rpsH*, signal recognition particle protein, threonyl-tRNA synthetase, *topA, valS *and 30S ribosomal protein S11; 41,617 bp). Bootstrap percentages after 2000 replications are shown. Because some genomes used in this study are not completely sequenced, for the comparison of 16S rRNA, MLSA and supertree, we used 16 genomes of vibrios. The genes used in MLSA and supertree were found only in these 16 genomes.

We selected both conserved and variable single copy genes belonging to different functional groups, from both chromosomes of vibrios and that have been used in several taxonomic studies [15,21,22,24,25,51]. Phylogenetic trees based on 16S rRNA gene sequences, MLSA and the supertree approach were constructed using the ML (Figure 4), MP (see Additional file 2; Figure S1) and NJ methods (see Additional file 3; Figure S2). The trees based on 16S rRNA gene sequences, MLSA and supertree showed similar topology in the three methods. Bootstrap analysis indicated that, most branches were highly significant. The phylogenetic reconstruction indicated a clear separation of groups (*i.e*. genera) within the vibrio clade. The genera *Photobacterium *and *Aliivibrio *were clearly separated from the genus *Vibrio*. The sister species of vibrios, *V. cholerae-V. mimicus*, *V. parahaemolyticus-V. alginolyticus *were separated from each other in the MLSA and supertree approaches in all three phylogenetic methods. These pairs of species had almost identical 16S rRNA gene sequences (≥ 99% sequence identity) though. Slight grouping differences were observed. *V. alginolyticus *appeared to be at the outskirts of the *V. parahaemolyticus *branch in the MLSA tree while in the supertree *V. alginolyticus *appeared at the outskirts of the *V. harveyi *branch in the three phylogenetic methods, simply because the number of genes used for each analysis was different. The difference may be due to different molecular clocks of the different genes. In the ML analysis, *V. vulnificus *appeared between *V. cholerae *and the vibrio core group. In all three phylogenetic methods, MLSA and supertree had the same taxonomic resolution to discriminate between species.

### Average amino acid identity (AAI)

The percentage of identity of putative orthologous protein-encoding genes detected in the pairwise comparison is shown in Figure 3 (and see Additional file 4; Table S2). The identity of protein-encoding genes between different genera of vibrios varied considerably. The mutual AAI for the pairs *Vibrio *and *Photobacterium*, *Vibrio *and *Aliivibrio*, and *Photobacterium *and *Aliivibrio *were at most 67%, 70% and 68%, respectively. The AAI within the genus *Phobacterium *(represented by *P. angustum S14 *and *P. profundum *SS9) was only 73%, whereas the AAI within the genus *Aliivibrio *was 85%. The AAI within the genus *Vibrio *varied between 70 and 91%. The *Vibrio *core group (i.e. *V. alginolyticus, V. campbellii, V. harveyi *and *V. parahaemolyticus*) shared at most 75% of their protein-encoding genes. The wider range of variation is explained by the higher number of representatives in the latter genus. The sister species *V. cholerae-V. mimicus*, *V. harveyi-V. campbellii*, *V. parahaemolyticus-V. alginolyticus *shared 90-91% AAI, whereas the intra-species AAI in *V. cholerae *varied between 98 and 99.5%. *Vibrio *sp. EX25 and *V. alginolyticus *40B had 95% identity, suggesting that EX25 belongs to the species *V. alginolyticus*. The rather low AAI within the species *V. harveyi *(*i.e*. 90%) may be due to the incomplete genome sequences and to unresolved taxonomic issues. *V. harveyi-like *1DA3 had 70% DDH in previous experiments and formed a separate genomic group on the basis of molecular fingerprinting [52].

### Dinucleotide relative abundance values (ρ*) and species-especifc genome signatures [δ*(*f*,*g*)]

ρ* values were in the normal range for all dinucleotides in all taxa investigated except for CG (over-represented in almost all genomes except in *V. campbellii*, *V. harveyi*, *V. shilonii*, *V. splendidus *and *Vibrio *sp. MED222) and TA (under-represented in almost all genomes except in *V. shilonii, P. angustum, P. profundum, A. fischeri *and *A. salmonicida*) (data not shown). The genomic dissimilarity value [δ*(*f*,*g*)] of the genus *Vibrio *towards the genera *Photobacterium *and *Aliivibrio *was 38-66 and 35-59, respectively. The δ*(*f*,*g*) value between the genera *Photobacterium *and *Aliivibrio *were in the range of 20 to 34. δ*(*f*,*g*) values within the genera *Alliivibrio *and *Photobacterium *were 26.5 and 13, respectively.

*δ*(*f*,*g*) values within each vibrio species were between 1 and 4, whereas the interspecies *δ*(*f*,*g*) were between 10 and 61 (Figure 3 and see Additional file 5; Table S3). Thus, the interspecies value was higher than the intergenus value. The *δ*(*f*,*g*) values among the *Vibrio *core group members and *V. cholerae/V. mimicus *were at least 38. The *δ*(*f*,*g*) values between the sisters species *V. cholerae-V. mimicus, V. harveyi-V. campbellii *and *V. parahaemolyticus-V. alginolyticus *were 14, 13 and 17, respectively. As vibrio species contain two chromosomes (one larger ca. 2.9 Mb and one smaller chromosome ca. 1.0 Mb) we also calculated the *δ*(*f*,*g*) between the two chromosomes of the same strain. The *δ*(*f*,*g*) values of the intragenomic comparison of the two chromosomes of complete vibrio genomes were between 10 and 18. The two chromosomes are essential for the cell survival and persistence, but yet they showed distinct patterns, suggesting a high genomic plasticity.

### Codon usage bias

Overall codon usage bias was very similar among the vibrio species investigated (Table 1). There was little variation in *N*_c _among the different genomes, with *N*_c _ranging from 45 to 54. The *N*_c_within the genera *Vibrio*, *Photobacterium *and *Aliivibrio *were 51-54, 48-51, and 45-48, respectively. Sister vibrio species had similar *Nc *values. Thus, there was not a clear differentiation of closely related taxa using the *Nc*.

**Figure 5:**
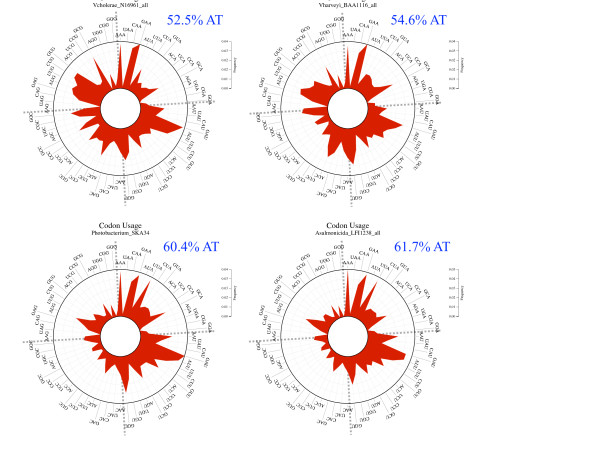
**Codon usage rose plots for four representatives of different vibrio genera**. The vibrios with a higher AT content (bottom rose plots) have a codon usage bias of A or U at the third position of the codon. The frequency scale is represented at the right side of the rose plots.

The rose plot shows the difference of codon usage for representative vibrios. The frequency of each codon is plotted in red. Distinguishable signatures for each genus are apparent (Figure 5). For instance, the genus *Vibrio *represented by *V. cholerae *and *V. harveyi *do not have a preferential codon usage, while the genera *Photobacterium *and *Alliivibrio *use codons that tend to end in either A or U. For instance, the frequency of UUA in *Photbacterium *and *Aliivibrio *genomes is higher than in *Vibrio *genomes, while the frequency of GCG is higher in *Vibrio *than *Photobacterium *and *Aliivibrio*.

## Discussion

### Genomic taxonomy

This study aimed at providing the underpins for the establishment of an online genomic taxonomy of vibrios. The methods used to extract taxonomic information from vibrios genomes are freely available in the web, have complementary taxonomic resolutions and are all amenable to automation for species and genera identification. Species identification is the major goal of microbial taxonomy. The identification of closely related sister species *V. cholerae *- *V. mimicus*, *V. alginolyticus *- *V. parahaemolyticus*, and *V. harveyi *- *V. campbellii *were evident in our study. The methods with the higher resolution for species and genera identification were, in order, MLSA, supertrees, and AAI. Karlin's genomic signature (*δ*(*f*,*g*)) performed well for species identification, whereas Nc appeared to be useful for differentiating genera.

### AAI

According to our analyses the AAI is one of the most useful genomic features for figuring out vibrio taxonomy. With the ever growing number of whole-genome sequences, this new method could be incorporated in a future re-valuation of the bacterial species definition. It is important to bear in mind that the number of orthologous genes shared between species depends on genome size and phylogenetic relationship [53,54]. Vibrio genomes have a wide variation in genome size, varying from 4 to 6 Mb (Table 1). As the genome size may influence the AAI, possibly the cut-off for species delineation will vary slightly when additional vibrio species are analysed.

### Genome signature dissimilarity δ*(*f*,*g*))

Karlin's genomic signature dissimilarity δ*(*f*,*g*)) can be used for species identification in vibrios. Overall, the genomic signature of vibrios was more similar between closely related species than to distantly related species. However, species from different genera may have similar signatures. For instance, *V. mimicus *and *Photobacterium *spp. shared values of genomic signature dissimilarity in the range of 38-40, whereas *V. mimicus *and *V. splendidus *had a genomic signature dissimilarity of 57. *V. campbellii *and *Aliivibrio fischeri *had a genomic signature dissimilarity of 35, indicating that the taxonomic resolution of Karlin's genomic signature δ*(*f*,*g*)) is lower for discriminating genera. Similar results were found in other studies concerning the resolution of this type of signature [55]. Genome signatures alone have significant limitations when used as phylogenetic markers for higher taxonomic levels *e.g*. genera to phyla. Apparently, the primary limitation is the lack of divergence in some phylogenetically distant related species that could result from absence of molecular clock. The equilibrium between mutational biases and selective constraints results in equilibrium in the oligonucleotide composition of a genome. Similar genome signatures between phylogenetically distant related species could arise from coincidental convergence due to crowding of the genome signature space derived from dinucleotide frequencies, which may not capture sufficient information to differentiate between distant taxa *e.g*. genera to phyla.

### Concordance between the methods

The vibrio genome BLAST atlas was an useful tool for depicting compositional differences between genomes of different species. Using this tool, differences between the sister species *V. cholerae *and *V. mimicus *in terms of gene content and DNA features were observed. Overall there was a significant correlation between the different methods (Table 2), but some methods had a stronger evolutionary signal and different taxonomic resolution than others. For instance, AAI and supertree showed the closest correlation with MLSA. All methods, except 16S rRNA and codon usage, provided significant (*P *< 0.001; T test) taxonomic resolution for differentiation of species and genera of vibrios. In general, the taxonomic resolution of 16S rRNA and codon usage was restricted to differentiation of genera.

**Table 2:** Pearson correlation coefficient (expressed as percentage) between different methods

	**1**	**2**	**3**	**4**	**5**	**6**
1. 16S rRNA gene identity	100					
2. Identity in MLSA	86.5	100				
3. Identity in supertree analysis	91.1	98.4	100			
4. Average aminoacid identity (AAI)	85.9	97.7	96.9	100		
5. Karlin genome signature dissimilarity	71.5	85.3	82.3	84.9	100	
6. BLAST proteome identity	77.1	89.0	86.4	92.5	85.5	100

The AAI and the proteome matrix correlated well, yet the latter is measuring the fraction of proteins that are the same in both genomes, and the former is measuring the average identity of the amino acids of the proteins in those matches. In addition, AAI uses 40% amino acid identity and > 70% of the aligned length of a protein which is stricter than the settings used (50% identity-50% length) to construct the proteome matrix. Because the Karlin's genomic signature dissimilarity indexes genome wide variation, its phylogenetic resolution is distinct of individual genetic marker genes. In addition, this signature considers variation in both coding and non coding genomic regions. This may explain why the correlation between the signature and the gene sequence based methods obtained in this study is slightly lower.

### Towards a new species definition in vibrios

A new species definition is mandatory if one aims to establish an automatic identification of vibrios through a web-based server. So far, the 16S rRNA gene analysis has been applied for species definition and identification [2]. Its value for these purposes in vibrios is limited because of its low taxonomic resolution. This study showed enough WGS-based evidence to propose a new species definition in vibrios. In our hands, a vibrio species is defined as a group of strains that share > 95% DNA identity in MLSA and supertree gene sequence, > 96% AAI, ≤ 10 genome signature dissimilarity, and > 61% proteome identity. Strains of the same species and species of the same genus will form monophyletic groups on the basis of MLSA and supertree.

### Ecology and genomic features

Each *Vibrio *species appear to have a specific ecologic niche. Genomes exhibit diverse patterns of species-specific compositional bias, *i.e *GC content, GC and AT skews, codon bias, and mutation bias. The exact mechanisms that generate and maintain the genome signatures are complex, but possibly involve differences in species-specific properties of DNA replication and repair machineries [49,56]. In *Borrelia burgdoferi*, there is a bias related to the speed of the replication [57], whereas in Proteobacteria, DNA repair enzymes co-evolve with the genome signature [58]. The evolutionary distances between DNA repair and recombination orthologous enzymes (mainly those involved in the nucleotide excision repair system) were highly correlated with genome signature distances. On the other hand, there was a significantly lower correlation between the evolutionary distances of the structural and metabolic enzymes and genome signature.

Environmental temperature and oxygen appear to influence the GC content of bacteria [59]. The frequencies of AA, TA, and TT dinucleotides were higher than the frequencies of AT, GC, and CG dinucleotides in the vaccine strain of *Pasteurella multocida *compared to the virulent strain. Although the vaccine strain is cultured at higher temperature, its GC content is lower than the virulent strain. The AA + TT dinucleotide increased significantly in the vaccine strain, which may represent an adaptation to increased culturing temperature because AA/TT dinucleotides are conformationally very stable. Higher culturing temperature increases spontaneous hydrolytic deamination of cytosine and 5-methylcytosine which, in turn, tend to decrease GC content [60,61]. Deamination and methylation favour nucleotide changes from G and C to A and T in a variety of microbial genomes [62].

Horizontal gene transfer may influence the genomic features of vibrios. The three genetic processes that mediate HGT often occur in vibrios and may cause phenotypic variation [63,64]. Such variation may confound a phenotype based identification. Another interesting feature of vibrios genomes is the presence of two chromosomes. The intragenomic dissimilarity between the two chromosomes of each vibrio strain is higher than the genomic dissimilarity between chromosome I of two strains of the same species. For instance, *V. cholerae *N16961 chromossomes I and II genomic dissimilarity *δ*(*f*,*g*)) was 12 and *V. cholerae *N16961 chromossome I and *V. cholerae *O395 chromossome I was 1. The fact that the two chromosomes of vibrios are dissimilar, with chromosome II less conserved than the chromosome I might support the hypothesis that the chromosome II was acquired by horizontal gene transfer [50]. The chromosome II has only a few essential housekeeping protein coding genes. This chromosome might have been a megaplasmid acquired by an ancestor prior diversification of the vibrios. Nearly 1/6 of the chromosome II (ca. 150 Kb) corresponds to a superintegron, a rapidly evolving region specialized in capture and loss of genes, and gene expression [65]. It is important to highlight that the fact that the two chromosomes of the same strain are more dissimilar than the chromosomes of different strains does not hamper the use of genome signatures for identification. Chromosomes I of *V. cholerae *and *V. mimicus *have signature dissimilarity higher than the signature dissimilarity between chromosome I of two *V. cholerae *strains. The same holds true for the chromosome II.

**Figure 6:**
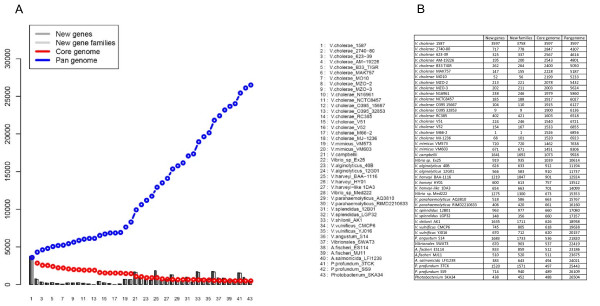
**Pangenome plot of vibrios**. Pangenome consists of panel A and panel B. The vibrio pangenome is around 26504 genes. *V. cholerae *has a pangenome of 6923 genes with clear increment of genes of its sister species *V. mimicus *(8306 genes).

The pangenome (N = 26504 genes) and the core genome (N = 488 genes) of all vibrios correspond to a vast reservoir of genetic diversity (Figure 6). The core genome of *V. cholerae *(N = 1520 genes) might represent the minimum set of genes that allow survival of the species in the environment, whereas the *V. cholerae *pangenome (N = 6923 genes) reflects the ability of this species to occupy different niches in the environment. The increase in the pangenome is due to new strain-specific genes which were found in each new *V. cholerae *strain analysed. Unique genes, e.g. the sensor kinase rscS, found in the *A. fischeri *allow this strain to occupy a specific niche in the environment (*i.e*. to colonize its squid host) [66]. The major toxin genes (CT and TCP) of *V. cholerae *toxigenic strains allow these strains to cause disease, but they were also found in *V. mimicus*. However, *V. mimicus *has not caused epidemics so far.

## Conclusion

The availability of new technologies for ultra-rapid whole genome sequencing and the development of concepts in comparative genomics will allow for rapid and reliable automatable identification of microbial isolates through a web-based server. The concept of an online electronic taxonomy based on whole genome features as illustrated in this study will improve microbial taxonomy. Environmental biodiversity surveys and ecologic studies on vibrios will also benefit from this new approach to identification. In this new context, traditional molecular approaches (*i.e*. DDH, MLSA, AFLP, rep-PCR) may still be useful for the screen of large collections of strains that will subsequently be used in whole genome based identification schemes.

## Abbreviations

DDH: DNA-DNA hybridization; AFLP: Amplified Fragment Lenght Polymorphism; HGT: Horizontal Gene Transfer; HR: Homologous Recombination; WGS: Whole Genome Sequencing; Tm: is the melting temperature of a double strand DNA molecule; ΔT_m_: is the difference between the Tm of a given double strand DNA molecule and the Tm of a hybrid of this molecule formed under controlled experimental conditions; CT: Cholera Toxin; TCP: Toxin Co-regulated Pilus.

## Authors' contributions

CCT carried out the computational analyses, phylogenetic and statistical analyses, analysed the results and wrote the manuscript. ACPV participated in the discussion and in the draft of the manuscript. RS and ATRV participated in the database construction and genomic anotation. TI carried out the pyrosequencing of the *V. mimicus *VM603 genome and helped writing the paper. NAJr obtained the vibrio samples and carried out the preliminary taxonomic identification of the genomes. DU carried out the pyrosequencing of the *V. alginolyticus *40B, *V. harveyi-like *1DA3 and *V. mimicus *VM573 genomes. DU and TV performed the BLAST atlas and matrix and the pangenome plot, and drafted the manuscript. FLT conceived the study, analysed the data and wrote the manuscript. All the authors have read and approved the final manuscript.

## Supplementary Material

Additional file 1 **Table S1. **BLAST matrix. The matrix lists the identity between proteomes of different strains of vibrios. The number of proteins and gene families in each genome are shown directly beneath the strain number. The hypotenuse (red) corresponds to the paralogs. The data provided the identity between proteomes of different strains of vibrios. The number of proteins and gene families in each genome are shown directly beneath the strain number. The hypotenuse (red) corresponds to the paralogs. Click here for file

Additional file 2 **Figure S1A-C. **Phylogenetic trees based on the maximum parsimony method using 16S rRNA gene, MLSA (*i.e*. *ftsZ, gyrB, mreB, pyrH, recA, rpoA and topA*; 10,141 bp), and supertree (*i.e*. aminopeptidase P, *alaS, aspS, ftsZ, gltX, gyrB, hisS, ileS, infB, metG, mreB, pntA, pheT, pyrH, recA, rpoA, rpoB, rpsH*, signal recognition particle protein, threonyl-tRNA synthetase, *topA, valS *and 30S ribosomal protein S11; 41,617 bp). Bootstrap percentages after 2000 replications are shown. Because some genomes used in this study are not completely sequenced, for the comparison of 16S rRNA, MLSA and supertree, we used 16 genomes of vibrios. The genes used in MLSA and supertree were found only in these 16 genomes. The data provided the phylogenetic relationship between vibrio strains Click here for file

Additional file 3 **Figure S2A-C. **Phylogenetic trees based on the neighbour-joining method using 16S rRNA gene, MLSA (*i.e*. *ftsZ, gyrB, mreB, pyrH, recA, rpoA and topA*; 10,141 bp), and supertree (*i.e*. aminopeptidase P, *alaS, aspS, ftsZ, gltX, gyrB, hisS, ileS, infB, metG, mreB, pntA, pheT, pyrH, recA, rpoA, rpoB, rpsH*, signal recognition particle protein, threonyl-tRNA synthetase, *topA, valS *and 30S ribosomal protein S11; 41,617 bp). Bootstrap percentages after 2000 replications are shown. Because some genomes used in this study are not completely sequenced, for the comparison of 16S rRNA, MLSA and supertree, we used 16 genomes of vibrios. The genes used in MLSA and supertree were found only in these 16 genomes. The data provided the phylogenetic relationship between vibrio strains Click here for file

Additional file 4 **Table S2. **Percentage of average amino acid identity (AAI) between vibrio species. Representative genomes were used for the calculations. The data provided the percentage of average amino acid identity (AAI) between vibrio species. Click here for file

Additional file 5 **Table S3. **Genomic dissimilarity [*δ*(*f*,*g*)] values between vibrio especies. Representative genomes were used for the calculations. The data provided the genomic dissimilarity [*δ*(*f*,*g*)] values between vibrio species. Click here for file
