# Supplementary figures and images for: Genomic taxonomy of vibrios
Source: BMC Evol Biol. 2009 Oct 27;9:258. doi: 10.1186/1471-2148-9-258 (PMC2777879; doi:10.1186/1471-2148-9-258)

79.8 %

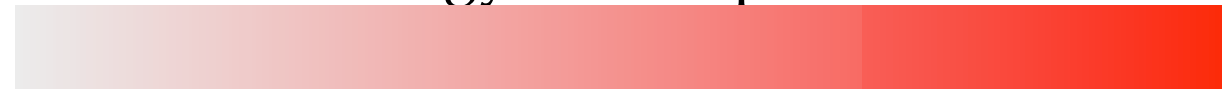

9.3 %

Supplement: Additional file 1 — Table S1. BLAST matrix. The matrix lists the identity between proteomes of different strains of vibrios. The number of proteins and gene families in each genome are shown directly beneath the strain number. The hypotenuse (red) corresponds to the paralogs. The data provided the identity between proteomes of different strains of vibrios. The number of proteins and gene families in each genome are shown directly beneath the strain number. The hypotenuse (red) corresponds to the paralogs. [file 1471-2148-9-258-S1.PDF]
