# Supplementary material for: Genomic taxonomy of vibrios
Source: BMC Evol Biol. 2009 Oct 27;9:258. doi: 10.1186/1471-2148-9-258 (PMC2777879; doi:10.1186/1471-2148-9-258)
Supplement: Additional file 2 — Figure S1A-C. Phylogenetic trees based on the maximum parsimony method using 16S rRNA gene, MLSA (i.e. ftsZ, gyrB, mreB, pyrH, recA, rpoA and topA; 10,141 bp), and supertree (i.e. aminopeptidase P, alaS, aspS, ftsZ, gltX, gyrB, hisS, ileS, infB, metG, mreB, pntA, pheT, pyrH, recA, rpoA, rpoB, rpsH, signal recognition particle protein, threonyl-tRNA synthetase, topA, valS and 30S ribosomal protein S11; 41,617 bp). Bootstrap percentages after 2000 replications are shown. Because some genomes used in this study are not completely sequenced, for the comparison of 16S rRNA, MLSA and supertree, we used 16 genomes of vibrios. The genes used in MLSA and supertree were found only in these 16 genomes. The data provided the phylogenetic relationship between vibrio strains [file 1471-2148-9-258-S2.PPT]

## Slide 1
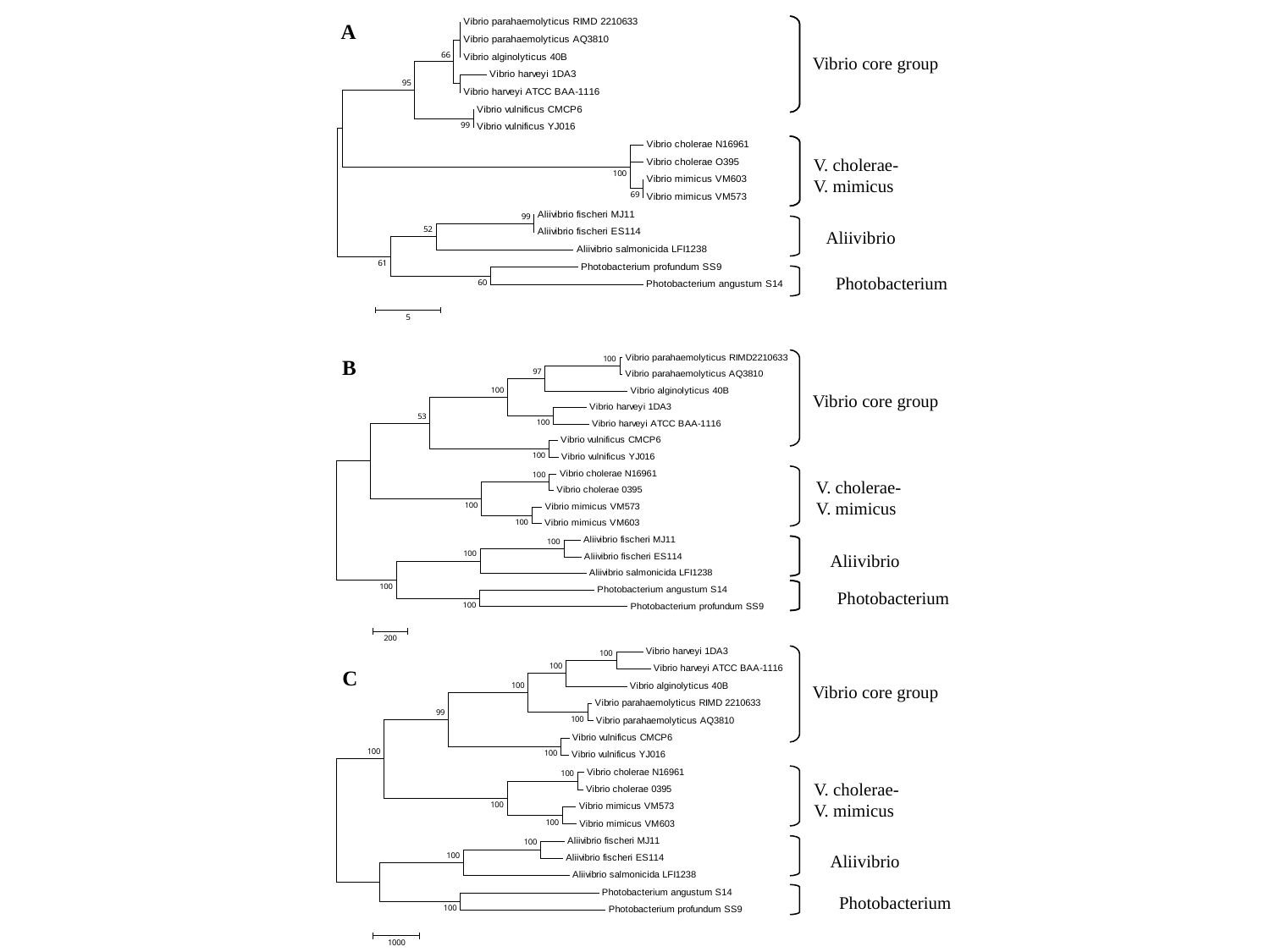

A
Vibrio core group
V. cholerae-
V. mimicus
Aliivibrio
Photobacterium
B
Vibrio core group
V. cholerae-
V. mimicus
Aliivibrio
Photobacterium
C
Vibrio core group
V. cholerae-
V. mimicus
Aliivibrio
Photobacterium
