# Supplementary material for: Genomic taxonomy of vibrios
Source: BMC Evol Biol. 2009 Oct 27;9:258. doi: 10.1186/1471-2148-9-258 (PMC2777879; doi:10.1186/1471-2148-9-258)
Supplement: Additional file 4 — Table S2. Percentage of average amino acid identity (AAI) between vibrio species. Representative genomes were used for the calculations. The data provided the percentage of average amino acid identity (AAI) between vibrio species. [file 1471-2148-9-258-S4.DOC]

| **Table 1. AAI (additional file 1)** |  |  |  |  |  |  |  |  |  |  |  |  |  |  |  |  |  |  |  |  |  |  |  |
| --- | --- | --- | --- | --- | --- | --- | --- | --- | --- | --- | --- | --- | --- | --- | --- | --- | --- | --- | --- | --- | --- | --- | --- |
| **Species name** | **1** | **2** | **3** | **4** | **5** | **6** | **7** | **8** | **9** | **10** | **11** | **12** | **13** | **14** | **15** | **16** | **17** | **18** | **19** | **20** | **21** | **22** | **23** |
| 1. V. alginolyticus 12G01 | - |  |  |  |  |  |  |  |  |  |  |  |  |  |  |  |  |  |  |  |  |  |  |
| **2. V. alginolyticus 40B** | 98 | - |  |  |  |  |  |  |  |  |  |  |  |  |  |  |  |  |  |  |  |  |  |
| 3. V. campbellii AND4 | - | 86 | - |  |  |  |  |  |  |  |  |  |  |  |  |  |  |  |  |  |  |  |  |
| 4. V. cholerae N16961 | 73 | 74 | 74 | - |  |  |  |  |  |  |  |  |  |  |  |  |  |  |  |  |  |  |  |
| 5. V. cholerae O395 | 73 | 74 | 74 | 99 | - |  |  |  |  |  |  |  |  |  |  |  |  |  |  |  |  |  |  |
| 6 .V. harveyi ATCC BAA-1116 | 87 | 87 | 91 | 74 | 73 | - |  |  |  |  |  |  |  |  |  |  |  |  |  |  |  |  |  |
| 7. V. harveyi HY01 | - | - | 90 | 72 | 72 | 96 | - |  |  |  |  |  |  |  |  |  |  |  |  |  |  |  |  |
| **8. V. harveyi-like 1DA3** | 87 | 88 | 90 | 73 | 73 | 90 | 92 | - |  |  |  |  |  |  |  |  |  |  |  |  |  |  |  |
| **9. V. mimicus VM573** | 74 | 74 | 74 | 91 | 91 | 74 | 72 | 73 | - |  |  |  |  |  |  |  |  |  |  |  |  |  |  |
| **10. V. mimicus VM603** | 74 | 74 | 74 | 91 | 91 | 74 | 72 | 73 | 98 | - |  |  |  |  |  |  |  |  |  |  |  |  |  |
| 11. V. parahaemolyticus RIMD2210633 | 90 | 90 | 86 | 74 | 74 | 87 | 86 | 87 | 74 | 74 | - |  |  |  |  |  |  |  |  |  |  |  |  |
| 12. V. parahaemolyticus AQ3810 | - | 90 | - | 74 | 74 | 87 | 85 | 87 | 74 | 74 | 98 | - |  |  |  |  |  |  |  |  |  |  |  |
| 13. V. shilonii AK1 | - | 72 | 72 | 71 | 71 | 73 | - | 72 | 71 | 71 | 73 | - | - |  |  |  |  |  |  |  |  |  |  |
| 14. V. splendidus 12B01 | - | 73 | 73 | 71 | 71 | 73 | - | 73 | 70 | 70 | 72 | - | 70 | - |  |  |  |  |  |  |  |  |  |
| 15. Vibrio. sp. EX25 | - | 95 | 84 | 72 | 72 | 86 | - | 86 | 72 | 72 | 91 | - | 71 | 71 | - |  |  |  |  |  |  |  |  |
| 16. Vibrio. sp MED222 | - | 76 | 75 | 73 | 73 | 76 | - | 76 | 73 | 73 | 75 | - | 72 | 91 | 73 | - |  |  |  |  |  |  |  |
| 17. V. vulnificus CMCP6 | - | 78 | 78 | 75 | 75 | 78 | - | 79 | 75 | 75 | 78 | - | 72 | 72 | 76 | 74 | - |  |  |  |  |  |  |
| 18. V. vulnificus YJ016 | - | 78 |  | 75 | 75 | 78 | - | 78 | 75 | 75 | 78 | - | - | - | - | - | 98 | - |  |  |  |  |  |
| 19. P. angustum S14 | - | 67 | 67 | 67 | 67 | 67 | - | 67 | 67 | 67 | 67 | - | 66 | 64 | 66 | 67 | 67 | - | - |  |  |  |  |
| 20. P. profundum SS9 | - | 67 | 67 | 66 | 66 | 66 | - | 66 | 66 | 66 | 67 | - | 66 | 65 | 65 | 67 | 67 | - | 73 | - |  |  |  |
| 21. A. fischeri ES114 | 69 | 70 | 69 | 69 | 69 | 69 | 68 | 69 | 68 | 68 | 69 | 70 | 68 | 67 | 68 | 70 | 69 | 69 | 68 | 68 | - |  |  |
| 22. A. fischeri MJ11 | - | - | - | 69 | 69 | - | - | - | 68 | 68 | - | - | - | - | - | - | - | - | - | - | 98 | - |  |
| 23. A. salmonicida LFI1238 | - | 70 | 69 | 69 | 69 | 68 | - | 69 | 69 | 69 | 70 | - | 67 | 67 | 67 | 69 | 69 | - | 67 | 67 | 85 | 85 | - |
