# Supplementary material for: Genomic taxonomy of vibrios
Source: BMC Evol Biol. 2009 Oct 27;9:258. doi: 10.1186/1471-2148-9-258 (PMC2777879; doi:10.1186/1471-2148-9-258)
Supplement: Additional file 5 — Table S3. Genomic dissimilarity [δ(f,g)] values between vibrio especies. Representative genomes were used for the calculations. The data provided the genomic dissimilarity [δ(f,g)] values between vibrio species. [file 1471-2148-9-258-S5.DOC]

| **Table 2. Genomic signature (additional file 2)** |  |  |  |  |  |  |  |  |  |  |  |  |  |  |  |  |  |  |  |  |  |  |  |
| --- | --- | --- | --- | --- | --- | --- | --- | --- | --- | --- | --- | --- | --- | --- | --- | --- | --- | --- | --- | --- | --- | --- | --- |
| **Species name** | **1** | **2** | **3** | **4** | **5** | **6** | **7** | **8** | **9** | **10** | **11** | **12** | **13** | **14** | **15** | **16** | **17** | **18** | **19** | **20** | **21** | **22** | **23** |
| 1. V. alginolyticus 12G01 | - |  |  |  |  |  |  |  |  |  |  |  |  |  |  |  |  |  |  |  |  |  |  |
| 2. V. alginolyticus 40B | 2 | - |  |  |  |  |  |  |  |  |  |  |  |  |  |  |  |  |  |  |  |  |  |
| 3. V. campbellii AND4 | - | 14 | - |  |  |  |  |  |  |  |  |  |  |  |  |  |  |  |  |  |  |  |  |
| 4. V. cholerae N16961 | 44 | 44 | 40 | - |  |  |  |  |  |  |  |  |  |  |  |  |  |  |  |  |  |  |  |
| 5. V. cholerae O395 | 44 | 44 | 40 | 1 | - |  |  |  |  |  |  |  |  |  |  |  |  |  |  |  |  |  |  |
| 6. V. harveyi ATCC BAA-1116 | 12 | 13 | 13 | 52 | 52 | - |  |  |  |  |  |  |  |  |  |  |  |  |  |  |  |  |  |
| 7. V. harveyi HY01 | 13 | 14 | 15 | 48 | 48 | 9 | - |  |  |  |  |  |  |  |  |  |  |  |  |  |  |  |  |
| 8. V. harveyi-like 1DA3 | 15 | 15 | 20 | 48 | 49 | 15 | 6 | - |  |  |  |  |  |  |  |  |  |  |  |  |  |  |  |
| 9. V. mimicus VM573 | 45 | 46 | 39 | 14 | 14 | 52 | 49 | 50 | - |  |  |  |  |  |  |  |  |  |  |  |  |  |  |
| 10. V. mimicus VM603 | 45 | 46 | 39 | 15 | 15 | 51 | 48 | 50 | 2 | - |  |  |  |  |  |  |  |  |  |  |  |  |  |
| 11 .V. parahaemolyticus RIMD 2210633 | 18 | 17 | 24 | 38 | 38 | 24 | 18 | 18 | 40 | 40 | - |  |  |  |  |  |  |  |  |  |  |  |  |
| 12. V. parahaemolyticus AQ3810 | - | - | - | 36 | 37 | 27 | 20 | 18 | 39 | 39 | 4 | - |  |  |  |  |  |  |  |  |  |  |  |
| 13. V. shilonii AK1 | - | 29 | 20 | 58 | 59 | 22 | 30 | 36 | 54 | 53 | 44 | - | - |  |  |  |  |  |  |  |  |  |  |
| 14. V. splendidus 12B01 | - | 18 | 19 | 59 | 59 | 10 | 19 | 24 | 57 | 57 | 33 | - | 14 | - |  |  |  |  |  |  |  |  |  |
| 15. Vibrio. sp. EX25 | - | 5 | 12 | 40 | 41 | 16 | 16 | 18 | 42 | 41 | 15 | - | 30 | 22 | - |  |  |  |  |  |  |  |  |
| 16. Vibrio. sp MED222 | - | 19 | 21 | 61 | 61 | 11 | 18 | 24 | 58 | 58 | 33 | - | 23 | 2 | 23 | - |  |  |  |  |  |  |  |
| 17. V. vulnificus CMCP6 | 38 | 38 | 33 | 16 | 16 | 45 | 41 | 42 | 19 | 18 | 31 | - | 52 | 53 | 34 | 54 | - |  |  |  |  |  |  |
| 18. V. vulnificus YJ016 | - | - | - | 16 | 16 | 45 | 41 | 42 | 19 | 18 | 31 | - | - | - | - | - | 1 | - |  |  |  |  |  |
| 19. P. angustum S14 | - | 54 | 47 | 51 | 51 | 58 | 62 | 66 | 46 | 47 | 61 | - | 45 | 55 | 52 | 56 | 58 | - | - |  |  |  |  |
| 20. P. profundum SS9 | - | 54 | 46 | 43 | 43 | 58 | 54 | 64 | 38 | 40 | 59 | - | 46 | 54 | 51 | 55 | 50 | - | 13 | - |  |  |  |
| 21. A. fischeri ES114 | 42 | 43 | 36 | 52 | 52 | 47 | 50 | 55 | 44 | 43 | 56 | 58 | 35 | 44 | 44 | 42 | 52 | 52 | 28 | 21 | - |  |  |
| 22. A. fischeri MJ11 | 42 | 43 | 35 | 51 | 51 | 47 | 50 | 54 | 43 | 43 | 58 | 57 | 35 | 43 | 44 | 41 | 51 | 51 | 27 | 20 | 1 | - |  |
| 23. A. salmonicida FLI1238 | - | 45 | 39 | 43 | 43 | 50 | 54 | 59 | 41 | 41 | 54 | - | 43 | 47 | 44 | 48 | 44 | - | 34 | 25 | 27 | 26 | - |
